# Comprehensive evaluation methods for translating BCI into practical applications: usability, user satisfaction and usage of online BCI systems

**DOI:** 10.3389/fnhum.2024.1429130

**Published:** 2024-06-05

**Authors:** He Pan, Peng Ding, Fan Wang, Tianwen Li, Lei Zhao, Wenya Nan, Yunfa Fu, Anmin Gong

**Affiliations:** ^1^Faculty of Information Engineering and Automation, Kunming University of Science and Technology, Kunming, China; ^2^Brain Cognition and Brain-Computer Intelligence Integration Group, Kunming University of Science and Technology, Kunming, China; ^3^Faculty of Science, Kunming University of Science and Technology, Kunming, China; ^4^Department of Psychology, School of Education, Shanghai Normal University, Shanghai, China; ^5^School of Information Engineering, Chinese People's Armed Police Force Engineering University, Xi’an, China

**Keywords:** online BCI system, usability of BCI system, user satisfaction of BCI system, usage of BCI system, analyzing and modeling for offline BCI data

## Abstract

Although brain-computer interface (BCI) is considered a revolutionary advancement in human-computer interaction and has achieved significant progress, a considerable gap remains between the current technological capabilities and their practical applications. To promote the translation of BCI into practical applications, the gold standard for online evaluation for classification algorithms of BCI has been proposed in some studies. However, few studies have proposed a more comprehensive evaluation method for the entire online BCI system, and it has not yet received sufficient attention from the BCI research and development community. Therefore, the qualitative leap from analyzing and modeling for offline BCI data to the construction of online BCI systems and optimizing their performance is elaborated, and then user-centred is emphasized, and then the comprehensive evaluation methods for translating BCI into practical applications are detailed and reviewed in the article, including the evaluation of the usability (including effectiveness and efficiency of systems), the evaluation of the user satisfaction (including BCI-related aspects, etc.), and the evaluation of the usage (including the match between the system and user, etc.) of online BCI systems. Finally, the challenges faced in the evaluation of the usability and user satisfaction of online BCI systems, the efficacy of online BCI systems, and the integration of BCI and artificial intelligence (AI) and/or virtual reality (VR) and other technologies to enhance the intelligence and user experience of the system are discussed. It is expected that the evaluation methods for online BCI systems elaborated in this review will promote the translation of BCI into practical applications.

## 1 Introduction

Brain-computer interface (BCI) is a new technology that subverts traditional human-computer interaction. It aims to directly establish a two-way closed-loop interaction channel between the brain and external devices, bypassing the peripheral nervous and muscular systems, to enhance the quality of life and work efficiency of patients, disabled people, and healthy individuals (Graimann et al., 2010; Allison et al., 2012; McFarland and Krusienski, 2012; Ramsey and Millán, 2020). BCI serves as a vital technology within neural engineering and rehabilitation engineering, harboring potential for medical applications. Despite having achieved significant milestones (Gao et al., 2021; Altaheri et al., 2023; Naser and Bhattacharya, 2023), the technology’s maturity is still nascent. It remains in the early stages of development, with a substantial gap to bridge before reaching practical applications (Ramsey, 2020).

To bridge the gap between BCI research and practical applications, researchers have proposed a gold standard for the online evaluation of BCI classification algorithms’ ability to generalize to new data (McFarland and Wolpaw, 2005; Krusienski et al., 2008; Mcfarland and Dean, 2012). However, comprehensive evaluation methods for the entire online BCI system remain scarce. In the online BCI system, while classification accuracy and bit rate are crucial metrics (Wolpaw et al., 2002), the paramount goal is to establish a system that is not only comprehensive but also user-friendly. This involves enhancing the system’s usability (Holz et al., 2013; Quek et al., 2013; van de Laar et al., 2013; Zickler et al., 2013; Riccio et al., 2015; Kübler et al., 2020), user experience (van de Laar et al., 2013), and user satisfaction (Zickler et al., 2011; Rupp et al., 2012; Holz et al., 2013, 2015; Quek et al., 2013; van de Laar et al., 2013; Pasqualotto et al., 2015; Vasilyev et al., 2017; Zander et al., 2017; Kübler et al., 2020).

**Figure 1:**
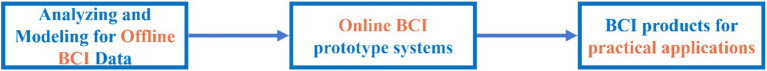
Leaps in the development of BCI systems.

It is the qualitative leap from analyzing and modeling for offline BCI data to constructing online BCI prototype systems, and then from prototype systems to real-world BCI products, as illustrated in Figure 1. Carefully considering BCI human factors engineering (Lu et al., 2021; Lyu et al., 2022) and adopting a user-centered approach to design and evaluate BCI systems (Zickler et al., 2009; Holz et al., 2013; Kübler et al., 2013, 2014, 2020; Liberati et al., 2015; Martin et al., 2018) are crucial for enhancing the usability and user satisfaction of BCI systems. However, the evaluation methods for translating BCI into practical applications have not received adequate attention within the BCI research and development community. Therefore, existing research is reviewed in the article, and more comprehensive evaluation methods for the entire online BCI system are detailed, aiming to promote the translation of BCI into practical applications. The purpose of this article is not to solve a specific problem of BCI. However, we believe that in addition to addressing key scientific and technological issues related to BCI, it is recommended to adopt the proposed comprehensive evaluation methods to evaluate the online BCI system. This approach should clarify what kind of BCI system the research community needs to develop.

**Figure 2:**
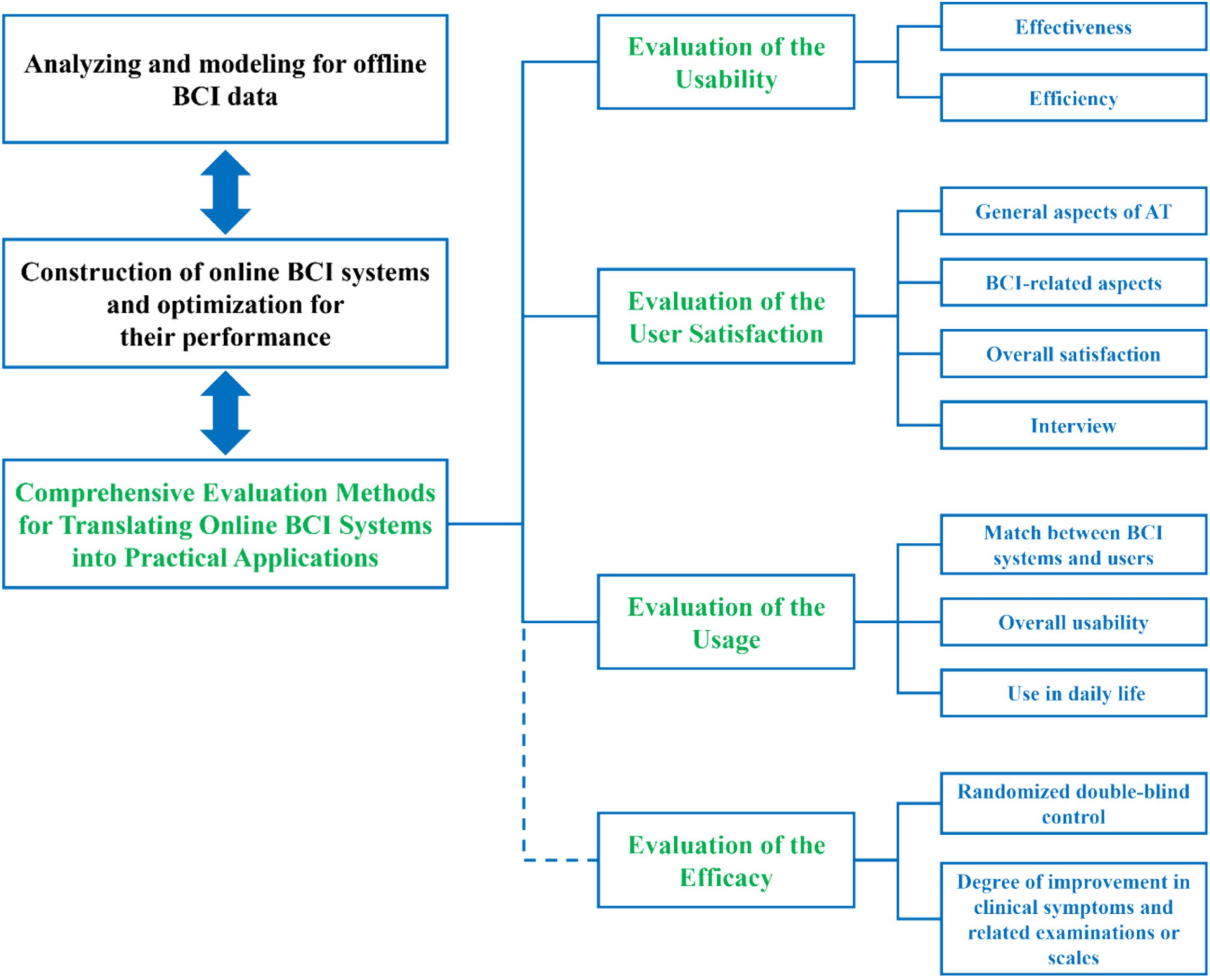
The schematic diagram of the comprehensive evaluation methods for translating BCI into practical applications.

The logical structure of this article is as follows: section two covers the qualitative leap from analyzing and modeling for offline BCI data to the construction of online BCI systems and optimization for their performance; section three covers the comprehensive evaluation methods for translating online BCI systems into practical applications, including the evaluation of the usability, user satisfaction, and usage of online BCI systems, as illustrated in Figure 2.

In addition to the above sections, section four is the discussions, and section five is the conclusions. In the discussion section of this paper, we elaborate on aspects that require attention. When translating BCI into practical applications, it is crucial to evaluate BCIs with different acquisition modalities (e.g., invasive, semi-invasive, and non-invasive) and paradigms [e.g., Motor Imagery (MI), Steady-State Visual Evoked Potentials (SSVEP), and P300] based on specific requirements and application scenarios. However, establishing evaluation methods for online BCI systems not only provides a consistent way to measure and compare the performance of different BCI systems but also offers clear goals and directions for R&D teams. Such evaluation methods can enhance the transparency and comparability of BCI technologies, thereby accelerating their optimization and improvement.

## 2 The qualitative leap from analyzing and modeling for offline BCI data to the construction of online BCI systems and optimization for their performance

To promote offline evaluation and comparisons of available BCI algorithms, the BCI research community has organized four international BCI data competitions from 2001 to 2008 (Sajda et al., 2003; Blankertz et al., 2004, 2006; Mcfarland and Dean, 2012). Although these data competitions offered useful suggestions for BCI algorithm improvement, they were focused on the analysis and modeling of offline BCI data, and their effectiveness still requires validation through online closed-loop testing (Mcfarland and Dean, 2012). The first live online BCI system competition took place at Tsinghua University in China in 2010, following the establishment of the BCI Research Award in 2009. Since then, similar competitions (such as the BCI-based brain-controlled robot competition at the annual World Robot Competition) have continued in China to promote the translation of BCI systems into practical applications.

Although offline evaluation can be used to identify a small number of promising alternatives, its biggest limitation is that it cannot evaluate the different impacts of different algorithms providing real-time feedback in online closed-loop operations (McFarland and Wolpaw, 2005; Krusienski et al., 2008). There is often a large discrepancy between the performance of models built from offline BCI data analyses and the closed-loop performance of online BCI systems, which needs to be submitted to online closed-loop testing. The results of the online closed-loop testing will lead to new offline analyses, which in turn will lead to new online studies, and this alternating iteration can effectively enhance the system’s performance (McFarland and Wolpaw, 2005; Krusienski et al., 2008). Online evaluation is the gold standard (McFarland and Wolpaw, 2005; Krusienski et al., 2008; Mcfarland and Dean, 2012). In the development process of BCI systems, analyzing and modeling for offline BCI data (initial BCI calibration or adjustment, including preliminary analysis and parameter optimization) are crucial steps for constructing online BCI systems. However, merely focusing on offline data analysis does not fulfill BCI’s ultimate objectives. Achieving a leap from offline modeling to the construction and performance optimization of online BCI systems is essential for translating BCI into practical applications and meeting the needs of the end-users of BCI.

### 2.1 Analyzing and modeling for offline BCI data

The aim of analyzing and modeling for offline BCI data is to reveal the brain signal features (neural encoding of user intentions) that correspond to the BCI paradigm, which includes external stimulation or mental tasks, and to establish and evaluate classification models, providing a foundation for constructing and optimizing online BCI systems.

Analyzing and modeling for offline BCI data primarily includes (1) BCI paradigm design, involving a carefully selected set of external stimulations or mental tasks tailored to specific brain signal acquisition techniques (Tai et al., 2024); (2) Raw brain signal acquisition, which includes setting up appropriate sampling rates and electrodes. Brain signals are acquired from recruited subjects during the execution of the designed BCI paradigm and saved for subsequent analyzing and modeling; (3) Brain signal preprocessing, such as filtering and artifact rejection to improve the signal-to-noise ratio (SNR); (4) Extracting and selecting time-frequency-spatial features with good discriminability for external stimulations or mental tasks, and discovering new features under innovative BCI paradigms; (5) Construction and optimization of intent decoding models based on machine learning or deep learning. It is crucial to choose the appropriate model structure according to the intended application scenarios and the nature of the selected brain signal features. Models such as linear discriminant analysis, support vector machines, deep neural networks, and linear regression are options. The acquired brain signal data is divided into training, validation, and/or test sets for model training through supervised learning and performance evaluation via cross-validation (e.g., accuracy and individual variability). Most BCI decoding models rely on supervised learning for model parameterization, which depends on the quality and quantity of the samples. It is noteworthy that optimizing each step is essential to achieve an effective model in analyzing and modeling offline BCI data.

Although analyzing and modeling for offline BCI data can be used to leverage the time and computational resources of offline analysis for complex data processing, evaluation, and comparison of algorithms to provide direction for the construction of online BCI systems and optimization for its performance. Research by Shenoy showed that numerous factors can contribute to changes in the statistical characteristics of the data between offline BCI calibration and online BCI control (e.g., non-stationarities in brain signal data, such as the mean and variance of the brain signal changing over time), and this change emphasizes the importance of testing and optimization for online closed-loop (Shenoy et al., 2006). These factors include technical factors such as variations in electrode placement or impedance; general user factors such as fatigue, frustration or motivation, user learning, large amounts of visual information that need to be processed in online operations and spontaneous variations (Shenoy et al., 2006).In addition, offline BCI data are typically collected without the use of neurofeedback, and the model built for offline BCI data analysis may be overfitting (over-learning), and lead to poor generalization and unstable performance (Billinger et al., 2013). However, the ability of a successful model to generalize to new data is a key requirement for BCI, because its online practical applications operation must use new data, these small sample data are unlabeled and can be used to re-parametrize models built from offline BCI data analysis online using semi-supervised or unsupervised learning (Mcfarland and Dean, 2012).

### 2.2 Construction of online BCI systems and optimization for their performance

**Figure 3:**
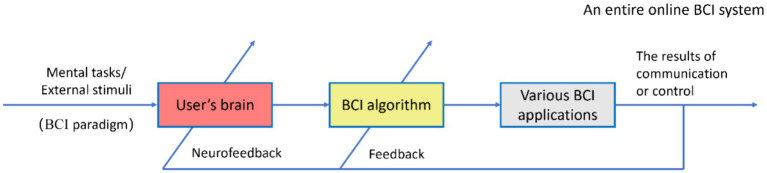
The schematic diagram for an entire online BCI system to be optimized and evaluated (Chen et al., 2024).

The construction of online BCI systems focuses on engineering implementations, including the real-time transmission of brain signal data, processing considerations (accounting for the computational demands of BCI algorithms), and the forms of neurofeedback used. Furthermore, it is crucial to recognize that the real-time closed-loop neurofeedback modulating the bidirectional co-adaptation between the user’s brain signals and BCI algorithms (Vidaurre et al., 2006; Krusienski et al., 2012; Perdikis et al., 2018; Wolpaw et al., 2020) presents the most significant challenge in constructing and optimizing the performance of online BCI systems. This aspect is also the primary distinction from the analysis and modeling for offline BCI data (without neurofeedback), as illustrated in Figure 3.

In Figure 3, the classification or control results of the BCI algorithm act on users in the form of neural feedback, which can regulate or affect the psychological activities of BCI users (e.g., strategies for executing psychological tasks), causing their brain state and signal characteristics to be changed, and to maintain or correct control instructions. This interaction can significantly diminish, or even nullify, the effectiveness of classification models developed through offline BCI data analysis. Consequently, BCI algorithms must dynamically adjust their parameters to accommodate changes in brain signal characteristics (Shenoy et al., 2006; Vidaurre et al., 2006; Wu and Hatsopoulos, 2008). Successful online BCI operation hinges on the effective interaction between two adaptive controllers (Taylor et al., 2002; Wolpaw et al., 2002; Krusienski et al., 2012; Mcfarland and Dean, 2012; Perdikis et al., 2018; Wolpaw et al., 2020): the user’s central nervous system (CNS) and the BCI algorithm responsible for brain signal processing and decoding. It is crucial to recognize that user and their brain constitute key components of the entire online BCI system. The arrow in Figure 3, traversing the user’s brain and the BCI algorithm, symbolizes their (bilateral) adaptive efforts to enhance and maintain the correlation between the user’s intentions and the overall output of the BCI system (Krusienski et al., 2012; Mcfarland and Dean, 2012; Billinger et al., 2013; Perdikis et al., 2018). The above are also BCI neuro-efficacy concerns of the user’s CNS interacting with the BCI system.

In Figure 3, users execute the BCI paradigm by accepting external stimuli, shifting attention, and performing mental tasks (e.g., sensory perception, cognition, and thought intentions), which generate brain signals related to the user’s intentions. Users develop skills for online BCI operation through operational training, thereby adapting to the BCI algorithm (Birbaumer et al., 2003). This process underscores the learnability and usability of BCI. Signals feedback to the BCI algorithm, including real-time brain signal characteristics, decoding outcomes or commands, control results, and scenes and objects controlled by BCI captured through machine vision, are utilized to refine the BCI algorithm for adaptability. This includes adaptive processes in brain signal processing, feature extraction, selection, and pattern classification algorithms, which are typically updated regularly by the system’s back-end through online machine learning.

When optimizing the online BCI system, each component (e.g., real-time brain signal collection and transmission, BCI paradigm optimization design, improving signal-to-noise ratio, and feature selection) needs optimization to improve the performance of the entire system. Translating online BCI prototype systems from the laboratory into practical applications should target typical scenarios while following the human-centred design principles and the activities involved in the system development lifecycle defined by the International Standards Organization (ISO) in 2010, as described in Tables 1, 2 (International Standards Organization, 2010; Holz et al., 2013; Kübler et al., 2013). A user-centered approach to designing and evaluating BCI systems (International Standards Organization, 2010; Holz et al., 2013; Kübler et al., 2013), which incorporates users into BCI systems development, is crucial for optimizing performance.

**Table 1:** Principles of Human-Centred-Design (HCD) defined by ISO (International Standards Organization, 2010; Holz et al., 2013; Kübler et al., 2013).

Number	Design principles
1	Include a clear understanding of user’s tasks and environmental requirements
2	Encourage an early and active involvement of users
3	Be driven and refined by user-centred evaluation
4	Iterate developmental stages for identification of optimal design solutions
5	Incorporate the whole user experience
6	Encourage multi-disciplinary design

**Table 2:** Activities involved in system development lifecycle defined by ISO (International Standards Organization, 2010; Holz et al., 2013; Kübler et al., 2013).

Number	Activities
1	Understand and specify the context of use
2	Specify the user requirements
3	Produce design solutions to meet user requirements
4	Evaluate the designs against requirements

As mentioned before, the user’s brain is a key component of the entire BCI system, which requires that when evaluating and optimizing the online BCI system (prototype system), objective evaluation indicators and user-subjective evaluation scales need to be combined (Lu et al., 2021; Lyu et al., 2022) to comprehensively evaluate its usability and user satisfaction.

## 3 Comprehensive evaluation methods for translating online BCI systems into practical applications

### 3.1 Evaluation of the usability of online BCI systems

Currently, there is a substantial gap between online BCI prototype systems and their practical application products, with relatively low usability levels hindering their translation to actual applications, thus necessitating significant improvements. The user-centered approach to BCI design (Zickler et al., 2009; Kübler et al., 2013, 2014; Liberati et al., 2015; Martin et al., 2018; Lu et al., 2021) defines BCI usability as the extent to which a specific end-user can use a particular BCI product to achieve a specific goal within a defined environment (Holz et al., 2013; Kübler et al., 2014; Abiri et al., 2020; Branco et al., 2021; Colucci et al., 2021; Lyu et al., 2022). BCI usability includes both effectiveness and efficiency.

#### 3.1.1 Evaluation of the effectiveness of online BCI systems

Accuracy (ACC) is used to evaluate the effectiveness of online BCI systems that produce discrete outputs. It is the most commonly used and key evaluation metric for evaluating online BCI systems, which is used to measure the accuracy of user intention recognition. Its calculation is illustrated in equation (1) and can be evaluated after each session.

(1) ACC=H/N

Where *H* denotes the number of correct trials and *N* denotes the total number of trials.

The performance of the online BCI that produces a continuous output can be evaluated with a continuous metric, usually using the *r*-square (*r^2^*), *r^2^* represents the proportion of the variance of the difference between the real output and the correct output (prediction error) of the model in the total variance of the output (Mcfarland and Dean, 2012), as illustrated in equation (2).

(2) $$r^{2}=\frac{SSR}{SST}=\frac{SST-SSE}{SST}=1-\frac{SSE}{SST}$$

Where *SSR* represents the square sum of the difference between the predicted data and the mean value of the original data, *SST* represents the square sum of the difference between the original data and the mean value, and *SSE* represents the square sum of the difference between the corresponding points of the predicted data and the original data.

#### 3.1.2 Evaluation of the efficiency of online BCI systems

Evaluation of the efficiency of online BCI systems includes information transfer rate (ITR), utility metric, and mental workload (Kübler et al., 2014). ITR is an important metric for evaluating the efficiency of the system, and it is used to evaluate the efficiency of the system in many studies, but the evaluation of the mental workload is also important. If users use the system with a large mental workload, it will seriously affect the acceptability and satisfaction of the system.

##### 3.1.2.1 Information transfer rate

ITR is a common metric for online BCI systems evaluation, which refers to the amount of information transmitted by the system in unit time (such as 1 min) in Bits/min (Wolpaw et al., 2002). Its calculation is illustrated in equation (3), which can be evaluated after each session.

(3) $$ITR=\frac{60\left[\log_{2}N+P\log_{2}P+\left(1-P\right)\log_{2}\left(\frac{1-P}{N-1}\right)\right]}{T}$$

Where *N* denotes the number of targets, *P* denotes the accuracy, and *T* denotes the time required to output a command.

In calculating ITR, the key is to determine the three parameters *N*, *P,* and *T*. In the case of an online synchronized BCI system, for example, *N* is known, *P* needs to be tested online, and the number of tests affects the estimation of *P*. T may be fixed, and the target shifting time affects the estimation of *T.* With constant *T*, *P* usually decreases as *N* increases (Yuan et al., 2013).

##### 3.1.2.2 Utility metric

One method of utility metric is that the effectiveness or accuracy of the online BCI system is less than 50%, the BCI is less efficient and has no practical utility, at this time the ITR value is meaningless, making the ITR value 0. In the case where the accuracy is greater than or equal to 50%, the ITR value has practical significance and can be evaluated after each session (Zickler et al., 2013).

Another method for utility metrics is to consider that different instructions may result in different benefits and define the utility as the expected average benefit (for the user) over time (Dal Seno et al., 2009), as illustrated in equation (4):

(4) $$U=E\left[\lim_{T\to \infty }\frac{\int_{0}^{T}b\left(t\right)dt}{T}\right]$$

Where *b* (*t*) is the gain function, which takes positive or negative values depending on whether the choice at moment *t* conforms to (or contradicts) the user intention, and *T* is the time required to output an instruction.

##### 3.1.2.3 Mental workload

In an online BCI system, the user serves both as the source (via the central nervous system) that generates control signals (brain signals) and the operator of the system. Operating the BCI system imposes a certain level of cognitive and psychological load on the user’s brain, referred to as mental workload. This workload is influenced by various factors, including the nature of the BCI application, task complexity, and the user’s experience level. A user-satisfying BCI system should impose a lower mental workload, thereby enhancing the user’s experience and satisfaction (Lu et al., 2021). The NASA Task Load Index (NASA-TLX) scale is commonly utilized to assess the mental workload of the user manipulating the BCI, as illustrated in Table 3, and can be evaluated after each session/task (Riccio et al., 2015).

**Table 3:** Mental workload evaluation scale for user manipulating BCI (Hart and Staveland, 1988; Lu et al., 2021; Lyu et al., 2022).

Dimension	Description	Scoring standard (0 ~ 100)
Mental (physiological) needs	Manipulate the BCI to complete the mental activity required to complete the task, whether the task is difficult	The greater the need the higher the score
Physical (physiological) needs	The physical strength required to control the BCI to complete the task, whether the muscle is tension, and movement are relaxed	The greater the need the higher the score
Time requirement	Does the speed requirement for manipulating BCI to complete tasks make human feel nervous or panicked?	The greater the need the higher the score
Effort level	The level of effort required to control the BCI to complete the task	The greater the need the higher the score
Performance level	Whether the performance level of controlling BCI to complete the task is satisfactory	The greater the need the higher the score
Frustration level	The levels of depression and frustration about the effectiveness of BCI manipulation	The greater the need the higher the score

The NASA-TLX scale was used (Hart and Staveland, 1988).

### 3.2 Evaluation of the user satisfaction of online BCI systems

In addition to evaluating the usability of online BCI systems, it is also need to evaluate the satisfaction of online BCI systems from the perspective of end users. Although some studies have evaluated the usability of online BCI systems (Morone et al., 2015; Alazrai et al., 2019; Lyu et al., 2022), only a few studies have evaluated system satisfaction (Kübler et al., 2014; Lyu et al., 2022). Excellent system satisfaction is the ultimate goal of the user-centred design of BCIs, and poor system satisfaction can severely affect the promotion of BCI system applications, so it is important to evaluate and improve the satisfaction of online BCI systems. Evaluation of the user satisfaction of online BCI systems mainly includes evaluation for general aspects of assistive technology (AT), BCI-related aspects, overall satisfaction, and interview for satisfaction (Kübler et al., 2014, 2020).

#### 3.2.1 Evaluation of general aspects of AT of online BCI systems

Essentially, a BCI system represents a new type of AT, enabling users to use their brain signals to interact directly with peripherals, thereby improving their quality of life or productivity. Therefore, it is necessary to evaluate the general aspects of AT satisfaction with online BCI systems. User satisfaction of AT is used to assess the level of user satisfaction with a particular AT product or service, which is typically evaluated using Quebec User Evaluation of Satisfaction with Assistive Technology 2.0 (QUEST 2.0) at the end of prototype testing (Rupp et al., 2012; Holz et al., 2013).

#### 3.2.2 Evaluation of BCI-related aspects

Evaluation of satisfaction of BCI-related aspects mainly reflects user satisfaction across four metrics reliability, learnability, speed, and aesthetic design. At the end of the prototype testing, users evaluated the four metrics on five scales from “not at all satisfied,” “not too satisfied,” “more or less satisfied,” “quite satisfied,” to “very satisfied” (Zickler et al., 2011).

##### 3.2.2.1 Reliability

The reliability of the BCI system is the ability to continuously complete a specified function within the specified time and environment, namely, the probability of the system operating without faults, which can be measured in terms of mean failure rate or mean time between failures (MTBF). The mean failure rate, denoted by *λ* (Rausand and Hoyland, 2003; O'Connor and Kleyner, 2012; Ebeling, 2019; Lu et al., 2021), refers to the probability of failure per unit time for BCI products that have not yet failed, calculated as illustrated in equation (5).

(5) $$\lambda =\frac{M}{\left(\Delta t\times N\right)}$$

Where *M* denotes the number of products that failed during the working time, *N* denotes the total number of products, and Δ *t* denotes the working time. The mean time between failures reflects the time quality of the BCI product, which is an ability to reflect the product’s ability to maintain its functionality for a specified period, and is calculated as illustrated in equation (6), where *λ* denotes the mean failure rate (Rausand and Hoyland, 2003; O'Connor and Kleyner, 2012; Ebeling, 2019; Lu et al., 2021).

(6) $$\mathrm{MTBF}=\frac{1}{\lambda }$$

The reliability of a BCI system is affected by a variety of major factors, including the quality of brain signal acquisition, brain signal processing algorithms, the stability of the BCI system, the accuracy of system calibration, real-time performance, persistence, environmental factors, and user factors, etc. For example, in electroencephalogram (EEG)-based BCI, the main challenge or difficulty faced by online BCI systems in practical application scenarios is that the EEG signals are very weak (microvolt level) and highly susceptible to interference from external environmental factors. BCI research is usually carried out in a structured and controlled laboratory environment, however, the BCI system is used outside the laboratory in a variety of application scenarios, where the EEG signals are highly susceptible to interference from external environmental factors, and advanced technology is required to reduce the interference and ensure the stability and reliability of the system (Gao, 2012). Therefore, the stability and reliability of online BCI systems need to be tested and evaluated in various application scenarios. Therefore, the stability and reliability of online BCI systems must be rigorously tested and evaluated across various application scenarios.

##### 3.2.2.2 Learnability

The learnability of BCI systems refers to how long it takes users to learn to use them (Nielsen, 1994; Colucci et al., 2021). Users usually need to spend a certain amount of time and training to learn to use the BCI system, if the majority of users can quickly learn to use the system, it indicates good learnability; otherwise, the system is considered less learnable. The learnability of a BCI system is influenced by a variety of factors, including the design of the system’s graphical user interface (GUI) and neurofeedback training.

##### 3.2.2.3 Speed

The speed of a BCI system usually refers to the system’s response time, that is, the time it takes for the system to capture the user’s brain signals until the system performs a specific operation or task (Colucci et al., 2021). It includes the time required for data acquisition, signal processing and classification, communication and control, and neurofeedback conditioning. It is an important performance metric, especially when applications require real-time control. In contrast, ITR measures the amount of information transmitted per unit of time (Wolpaw et al., 2002).

##### 3.2.2.4 Aesthetic design

The aesthetic design of a BCI system refers to the aesthetic factors of user interface design and product appearance design (especially the appearance of the sensors that capture brain signals) when developing a BCI system product (Tractinsky et al., 2000; Norman, 2005; Colucci et al., 2021). Considering different users’ aesthetic preferences, the BCI system offers personalization options. The aesthetic design of the BCI system affects user acceptance, comfort, experience, and user satisfaction.

Besides these 4 aspects, the sensors used in online BCI systems greatly determine the user’s experience and acceptability. User satisfaction with BCI sensors is very important and can be evaluated in five dimensions: safety, comfort, aesthetic, ease of use, and overall satisfaction, with scores ranging from the lowest 1 to the highest 5, as illustrated in Table 4 (Lu et al., 2021; Lyu et al., 2022).

**Table 4:** An example of user satisfaction of the BCI sensor used in a certain experiment (Lu et al., 2021; Lyu et al., 2022).

The type of BCI sensor			Evaluation grade				
			Safety	Comfort	Aesthetic	Ease of use	Overall satisfaction
Sensor for non-invasive BCI	EEG sensor on scalp surface	Conductive gel electrode	5	3	3	3	3
		Physiological saline electrode	5	4	3	4	4
		Dry electrode	5	3	4	5	4
	NIRS sensor	Emitting and detecting probes	5	3	4	4	4
	MEG sensor	Non-contact sensor for measuring magnetic field strength	x	x	x	x	x
Other non-invasive BCI sensor		x	x	x	x	x
Sensor for invasive BCI	ECoG sensor	Platinum electrode array	x	x	x	x	x
	Intracortical sensor (Spikes, LFP)	Multi-electrode array	x	x	x	x	x
		Multi-site electrode	x	x	x	x	x
		Cone-shaped electrode	x	x	x	x	x
		Cone-shaped electrode	x	x	x	x	x
	Other invasive BCI sensor		x	x	x	x	x

NIRS (Near-Infrared Spectroscopy), MEG (Magnetoencephalography), ECoG (Electrocorticography), LFP (Local Field Potential).

##### 3.2.2.5 Evaluation of user experience of BCI

User experience of BCI is an important aspect of BCI user satisfaction. It is the user’s personal feeling and experience of using the BCI system. Applications like rehabilitation training systems combining BCI with VR, and BCI-controlled games, offer a user experience characterized by immersion (involvement and/or losing track of time), pleasure, engagement, and presence (in the case of a game, user experience being “in” the virtual world) (Van Baren, 2004; Jennett et al., 2008; Brockmyer et al., 2009). The evaluation of the BCI user experience helps to increase user acceptance of BCI, improve system performance, and increase pleasure. Observational analysis (observing and recording user behavior to provide objective-qualitative data), neurophysiological measurements (recording EEG signals, galvanic skin response (GSR), and electrocardiogram (ECG) when the user manipulates the BCI to provide objective quantitative data), interview (to provide subjective qualitative information), and questionnaires (to provide subjective quantitative information) can be used to evaluate for user experience of BCI (Mandryk et al., 2006; Gürkök et al., 2011).

The above satisfaction evaluation of general and BCI-related aspects of online BCI systems AT satisfaction can be found in the user satisfaction with assistive technology evaluation from Quest 2.0 and its expansion table (Colucci et al., 2021), as illustrated in Table 5. Items 1–12 in the table evaluate the comfort, size, ease of use, effectiveness, ease of installation and adjustment, safety, quality of service, weight, reliability, real-time (rapidity), ease of learning, and aesthetic of the BCI system, and the evaluations are classified into five grades of very satisfied, satisfied, average, dissatisfied, and very dissatisfied. Items 13–16 in the table can be used as metrics for evaluating the final BCI product used by the user, and the evaluations are carried out after the BCI system is implemented (Zickler et al., 2011; Kübler et al., 2014; Colucci et al., 2021; Lu et al., 2021; Lyu et al., 2022).

**Table 5:** BCI system satisfaction evaluation item (Colucci et al., 2021; Lu et al., 2021; Lyu et al., 2022).

Evaluation item	Evaluation item description
(1) How satisfied are you with the comfort level of your current BCI equipment?	What is the comfort level of the BCI sensor and the comfort level of mental tasks (SSVEP, P300, MI)?
(2) How satisfied are you with the size (length, width, and height) of the current BCI equipment?	Are the sizes of BCI sensors and amplifiers ultra-miniaturized or portable?
(3) How satisfied are you with the ease of use of the current BCI equipment?	Is BCI graphical user interface simple and easy to use, and are mental tasks easy to complete?
(4) How satisfied are you with whether the current use of BCI equipment can be assistive or its effectiveness?	How satisfied are you with the tasks accomplished by SSVEP-BCI P300-BCI MI-BCI?
(5) How satisfied are you with whether the current BCI equipment is easy to install and adjust?	Is the software and hardware of the BCI system easy to install and adjust? Specifically, it may include whether the sensor is easy to wear and adjust, the amplifier parameter setting, whether the BCI software is easy to install and set, and whether the BCI and the external device are easy to communicate with the interface.
(6) How satisfied are you with the safety of BCI equipment?	How safe is the invasive BCI sensor? How safe is the BCI control system? E.g., the obstacle avoidance ability of a brain-controlled wheelchair.
(7) How satisfied are you with the access channel and efficiency of BCI equipment?	Obtain BCI after-sales service channels and service efficiency, including whether BCI can be used by independent families, and minimize the dependence on BCI technical support.
(8) How satisfied are you with the weight of BCI equipment currently in use?	Are BCI sensors and amplifiers super light?
(9) How satisfied are you with the reliability of the current BCI equipment?	What is the ability of the BCI system to perform specified functions without failure in a certain period of time and under certain conditions, such as reliability, failure rate and mean time between failures?
(10) How satisfied are you with the response time of the current BCI equipment?	How fast is the BCI system? What is the specific ITR?
(11) How satisfied are you with the learnability for BCI?	Is the operation of the BCI system easy to learn? This includes whether the BCI graphical user interface (GUI) and mental tasks are learnability.
(12) How satisfied are you with the appearance of BCI equipment?	Are the graphical user interface (GUI) and sensors of the BCI system beautiful? For the BCI sensor: Is it concealed and does it match the visual aesthetic?
(13) How satisfied are you with the professional services of BCI equipment provided by medical staff?	For the clinical application of BCI, it is necessary to evaluate the professional service quality of medical staff.
(14) How satisfied are you with the robustness and durability of BCI equipment currently in use?	How robust are the BCI sensors and amplifiers?
(15) How satisfied are you with the maintenance service of BCI equipment currently in use?	What is the frequency of BCI system failure or maintenance and the quality of maintenance service? Including easy contact and maintenance efficiency.
(16) How satisfied are you with the follow-up BCI equipment consultation and tracking services provided by medical staff?	For the use of BCI in follow-up daily life, we need to evaluate the quality of follow-up service of medical staff.

Refer to the user satisfaction evaluation form for assistive technology Quest 2.0 and its expansion table (Colucci et al., 2021).

#### 3.2.3 Evaluation of overall satisfaction of online BCI systems

The satisfaction evaluation in Table 5 for the BCI system includes many items and is time-consuming, which makes it inconvenient to evaluate the satisfaction level of different users when they try the BCI prototype to complete different tasks (the same BCI product to complete tasks with different functions) during the rapid prototyping iteration process (Lu et al., 2021). A simple and fast visual analog scale (VAS) (Allison et al., 2012; Kübler et al., 2014) is often used to evaluate users’ usage of the system in overall satisfaction with online BCI systems. As illustrated in Table 6, the satisfaction levels of different users when controlling the BCI to complete various tasks are rated from ‘dissatisfied (1)’ to ‘absolutely satisfied (10)’, with evaluations conducted after each session (Holz et al., 2015a,b).

**Table 6:** Visual Analog Scale (VAS) (Allison et al., 2012; Kübler et al., 2014).

Task	User 1			User 2			User…		
	Task 1	Task 2	…	Task 1	Task 2	…	Task 1	Task 2	…
Satisfaction 1 ~ 10									

#### 3.2.4 Interview for the satisfaction of online BCI systems

Interview for the satisfaction of BCI online systems refers to interviews with users of the system to find out how satisfied they are with using the BCI system (Ramsey and Millán, 2020). Interviews can be conducted end of prototype testing or after the sale of BCI products using semi-structured or free-form questionnaires (Kübler et al., 2014; Vasilyev et al., 2017; Ma et al., 2023).

### 3.3 Evaluation of the usage of online BCI systems

#### 3.3.1 Evaluation of the match between BCI systems and users

Evaluation of the match between BCI system (production) and user can be used the questionnaire Assistive Technology Device Predisposition Assessment (ATD-PA) Device Form -Initial Consumer and Professional (Holz et al., 2015a). It is a set of questionnaires based on the Matching Person and Technology Model (MPT) (Kübler et al., 2014; Ma et al., 2023), and it comprises 12 items (see Table 7), as illustrated in Table 7 (Zickler et al., 2013; Corradi et al., 2017). It addresses the primary users (end-users and consumers) and secondary users (professionals, including professional users/AT experts/researchers) to rate their predisposition for using the BCI system under consideration.

**Table 7:** Evaluation form of the match between BCI systems and users (Gürkök et al., 2011; Zickler et al., 2013; Corradi et al., 2017).

Item	ATD-PA device form
1	Will the BCI system help me to achieve my goals?
2	Will the BCI system benefit me and improve my quality of life?
3	Can I be confident that I know how to use the BCI system and its various features?
4	Will I feel more secure (safe, confident) when using the BCI system?
5	Will the BCI system fit my accustomed routine?
6	Do I have the capabilities and stamina to use the BCI system without discomfort, stress, and fatigue?
7	Is there support, assistance and accommodations for successful use of the BCI system?
8	Will the BCI system physically fit in all desired environments (car, living room, etc.)?
9	Will I feel comfortable using the BCI system around family?
10	Will I feel comfortable using the BCI system around friends?
11	Will I feel comfortable using the BCI system at work?
12	Will I feel comfortable using the BCI system around the community?

In Table, it has to be rated on a 5-point Likert scale from 1 to 5. Users have the option to indicate a “0” if the item is not applicable. The total score was calculated by averaging all item scores. The highest score is 5.0. A score between 4.0 and 5.0 indicates a good match of users and the BCI system, scores below 4.0 indicate that the match could be improved, and a score of 3 or less indicates a risk of system non-use (Zickler et al., 2013; Kübler et al., 2014; Holz et al., 2015a; Corradi et al., 2017).

#### 3.3.2 Evaluation of the overall usability of BCI systems

The overall usability of BCI systems can be evaluated using the System Usability Scale (SUS) after prototype testing (Pasqualotto et al., 2015; Zander et al., 2017). The SUS contains 10 items, with a global subjective assessment of overall usability. Each item’s score ranges from 0 to 100 points, as illustrated in Table 8, where higher scores indicate better overall usability of the BCI system, and a score of 70 has been suggested as the acceptable minimum (Brooke, 1996; Bangor et al., 2008; Pasqualotto et al., 2015).

**Table 8:** Evaluation form of the overall usability of the BCI systems (Brooke, 1996; Bangor et al., 2008).

Item	System Usability Scale (SUS)
1	I think that I would like to use the BCI system frequently.
2	I found the BCI system unnecessarily complex.
3	I thought the BCI system was easy to use.
4	I thought that I would need the support of a technical person to be able to use the BCI system.
5	I found that the various functions in the BCI system were well integrated.
6	I thought that there was too much inconsistency in the BCI system.
7	I thought most people could learn to use the BCI system very quickly
8	I thought the BCI system very inconvenient to use.
9	I felt confident using the BCI system.
10	I needed to learn a lot of things before using the BCI system.

#### 3.3.3 Evaluation of the use in daily life of online BCI systems

The ultimate proof for use in daily life of BCI systems is its actual use (Kübler et al., 2014), which can be investigated by interviewing specific BCI users on four issues in use, as illustrated in Table 9, the four issues investigated are the ones that need to be considered for the translation of BCI technology into practical applications (Vaughan et al., 2012; Ramsey and Millán, 2020; Vaughan, 2020; Ma et al., 2023).

**Table 9:** Evaluation of the use in daily life of online BCI systems (Bangor et al., 2008; Vaughan et al., 2012; Ramsey and Millán, 2020; Vaughan, 2020).

Item	Content of evaluation	Level of evaluation
1	Can the people who need a BCI use one?	Cannot\basically can\can
2	Is the personalized BCI suitable for long-term independent use?	Not suitable\suitable
3	Does the personalized BCI get used, and how does it get used?	Not used, less used, often used How to use it?
4	Does the personalized BCI improve the user’s lives? (Vaughan et al., 2012)	Not improvement\less- improvement \improvement

## 4 Discussion

Different researchers may have different methods for evaluating online BCI systems. Besides evaluating classification accuracy and bit rate, it is also necessary to perform a comprehensive evaluation of online BCI systems, including evaluating user satisfaction, usage, and efficacy.

### 4.1 Challenges faced in the evaluation of the usability and user satisfaction of online BCI systems

The online BCI system requires user involvement in its semi-automated loop, where users not only act as the source of signals for system communication and control (via their central nervous system) but also directly interact with it as operators. This direct interaction presents challenges for assessing the system’s usability and satisfaction. Users must produce brain signal features recognizable by the BCI algorithm, yet ensuring the generation of such features is challenging. It necessitates that BCI developers innovate paradigms (Tai et al., 2024) and neurofeedback adjustment strategies tailored for users, encompassing the feedback of suitable neural signals and their presentation. Furthermore, users often need to acquire skills to effectively use and derive benefits from BCI, which involves assessing the BCI’s efficacy.

Therefore, it is necessary to evaluate specific end-users utilizing the specific BCI system to achieve specific goals within specific environments (Kübler et al., 2014). The focus of the evaluation metrics mentioned in this article varies, for instance, some BCI applications (such as control applications (Leeb et al., 2013; Edelman et al., 2019)) typically require high accuracy and real-time/timeliness. On the other hand, other BCI applications [such as active rehabilitation training (Cui et al., 2021; Shen et al., 2022)] may need to capture the trainee’s attention and provide rewards. If the purpose of the BCI system is to foster brain plasticity, evaluating changes in the connectivity and function of relevant brain regions becomes essential.

### 4.2 Evaluation of the efficacy of BCI

In addition to the evaluation of the usability and user satisfaction of online BCI systems, the evaluation of the efficacy of BCI is also essential. BCI efficacy pertains to the functional or therapeutic benefits it provides to users (including patients), or the outcomes or anticipated effects it produces. BCI efficacy encompasses monitoring (the brain’s state), replacement (outputs lost due to injury or disease), improvement/restoration (enhancing disease symptoms or restoring functions), enhancement (function improvement and expansion), and supplementation (adding brain control methods). However, methods for evaluating the efficacy of BCI in treating or rehabilitating CNS-related diseases/disorders remain unstandardized. Collaboration among BCI clinical translation researchers or producers, clinicians, and patients is imperative to objectively assess the medical applications’ efficacy and avoid subjective evaluations or unwarranted hype (Innovation Collaboration Platform for Artificial Intelligence Medical Devices, 2023). Clinically, the efficacy of BCI is evaluated using a randomized double-blind controlled method, considering both the degree of symptom improvement and clinically relevant examinations or scales, including objective measures by medical instruments (like muscle strength and electromyography) and subjective scales. For instance, Biasiucci A has demonstrated that BCI-functional electrical stimulation (BCI-FES) therapy promotes significant functional recovery and purposeful plasticity through conditional activation of natural efferent and afferent pathways (Biasiucci et al., 2018). Identifying the most suitable application scenarios for online BCI systems in various fields requires ongoing research and validation to optimize their applicability and effectiveness.

### 4.3 What methods can be used to improve the usability and user satisfaction of online BCI systems?

In addition to the evaluation of the usability and user satisfaction of online BCI systems, what other methods can be used to improve these two metrics? Currently, the usability and user satisfaction of online BCI systems are not high. One of the reasons may be that the BCI system is less intelligent. In essence, the BCI system is driven by a set of external stimuli or mental tasks carefully selected/designed by the developer in advance. When using BCI, users can manipulate BCI according to the BCI paradigm but cannot do whatever they want; otherwise, it will be difficult for the BCI system to recognize their intentions. At present, the user experience of the BCI system is poor, the brain-computer interaction content is not rich, and the human-computer interaction is monotonous or boring.

#### 4.3.1 Integration of BCI and AI

**Figure 4:**
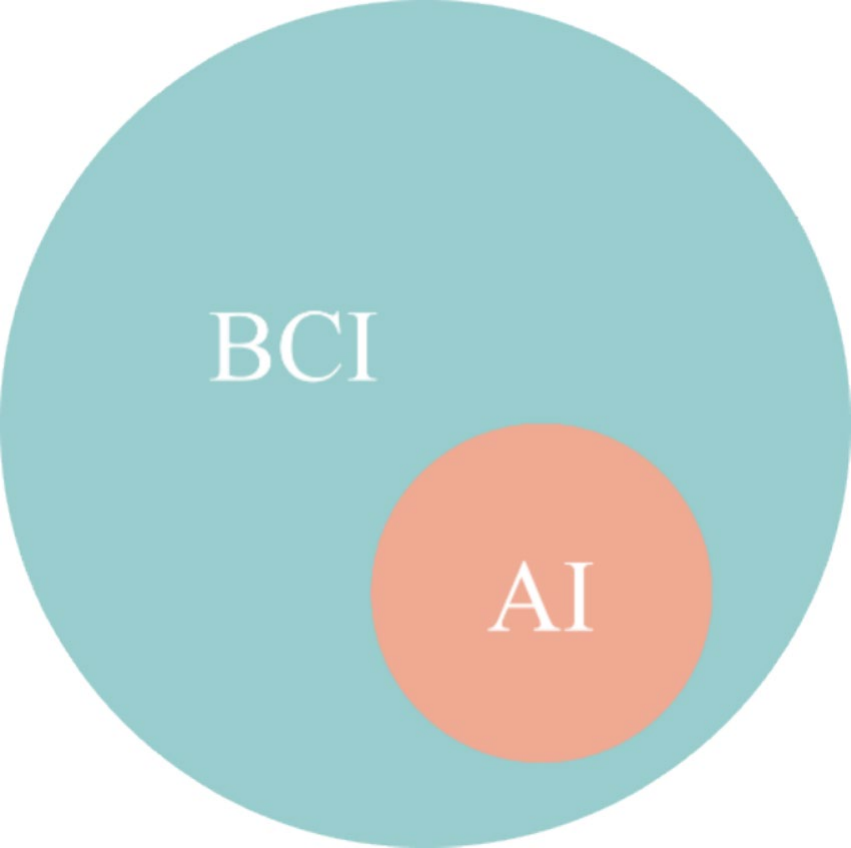
The schematic diagram for the integration of BCI systems and AI to improve intelligence (Lu et al., 2021; Lyu et al., 2022; Innovation Collaboration Platform for Artificial Intelligence Medical Devices, 2023).

Due to the limited intelligence of BCI, it can be integrated with AI technology to add intelligent elements. For example, advanced and effective machine learning (specifically, deep learning) can be used for brain signal analysis and adaptive machine learning in BCI systems to improve decoding accuracy and ITR (Willett et al., 2021; Metzger et al., 2023; Willett et al., 2023). Communicative BCI can interact with natural language models to enrich communication content, and BCI systems can be combined with computer/machine vision technology (constructed intelligent environments) to improve the intelligent interactivity between users and the environment. Figure 4 illustrates the integration of BCI systems and AI to improve intelligence (Lu et al., 2021; Lyu et al., 2022; Innovation Collaboration Platform for Artificial Intelligence Medical Devices, 2023).

#### 4.3.2 Integration of BCI and VR

Given the current BCI system’s poor user experience, integrating it with VR could enhance its operability and interactive experience. VR technology allows for the visualization and user control of implicit mental tasks, significantly enhancing the user experience for BCIs that rely on implicit mental tasks. For example, VR can vividly guide BCI users to improve motor imagery quality.

**Figure 5:**
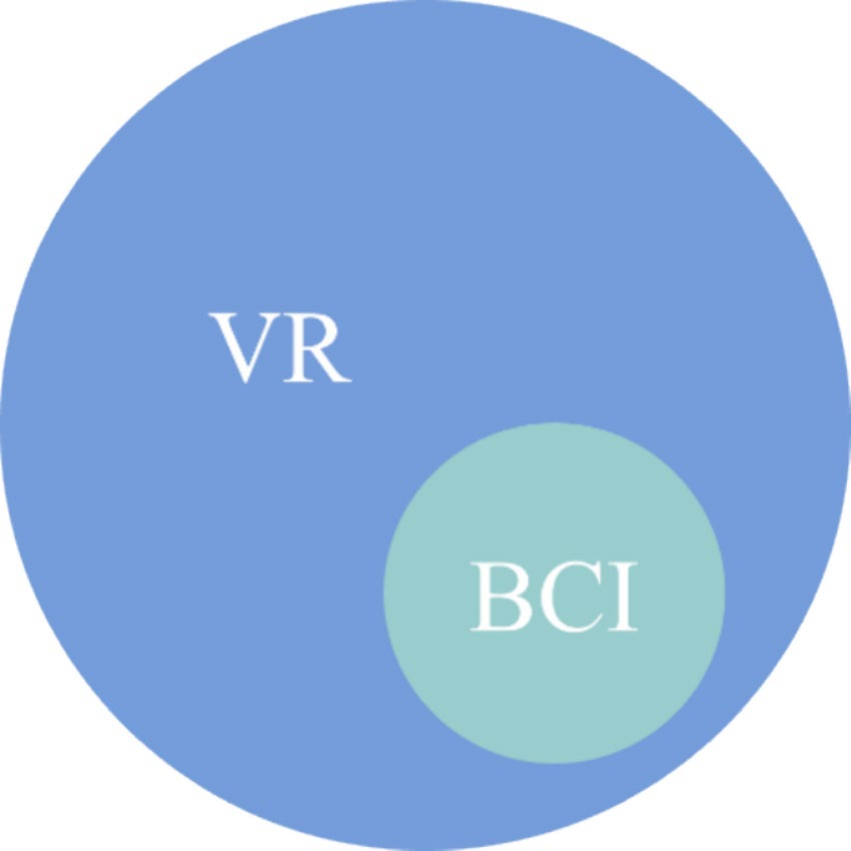
The schematic diagram for the integration of BCI systems and VR to improve user experience.

BCI can also be integrated with VR-based games to enhance users’ attention and give timely rewards, thereby allowing BCI users to effectively regulate brain activity and signals, fostering neuroplasticity, and improving the immersion and fun of using BCI (Currently, the immersion and fun of using BCI are poor, and the experience is not high). Additionally, VR creates an immersive environment for training and control, enhancing the BCI system’s learnability. Therefore, combining BCI with VR-based simulation training effectively boosts users’ engagement and interest in performing psychological tasks and substantially improves the effectiveness of BCI-based rehabilitation training. Figure 5 illustrates the integration of BCI systems and VR to improve user experience.

#### 4.3.3 Integration of BCI with AI and VR

**Figure 6:**
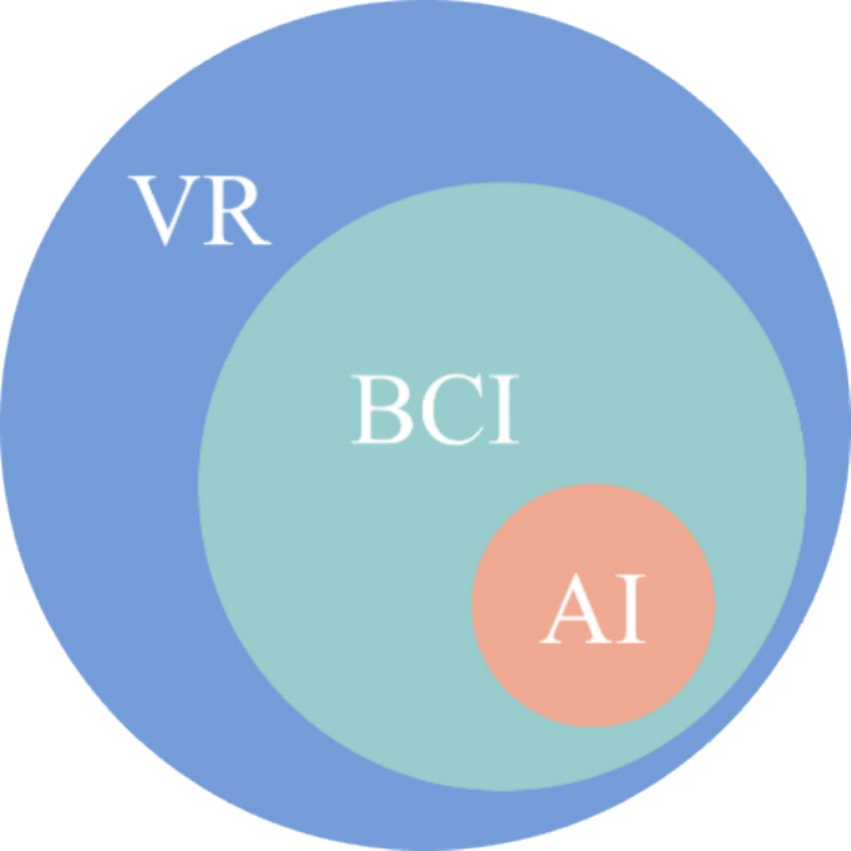
The schematic diagram for the integration of BCI systems with AI and VR at the same time.

To simultaneously improve the intelligence and user experience of the BCI system, the BCI can be integrated with AI and VR at the same time to create smarter and richer interactive scenarios for users to control BCI. Figure 6 illustrates the integration of the BCI system with AI and VR.

### 4.4 What barriers are faced in translating different collection methods and different paradigms of BCI into practical applications?

Translating invasive, semi-invasive, and non-invasive BCI into practical applications encounters several common barriers: (1) Technical complexity. BCI systems involve complex signal acquisition, processing, and decoding processes, necessitating highly accurate and real-time technical support; (2) Adaptability to users. The individual differences in users’ brain activity patterns require BCI systems to be highly adaptable and personalized; (3) Cost issues. The expenses related to system development and user equipment pose significant barriers to application promotion.; (4) Ethics and privacy. BCI technology entails direct access to human brain activity, raising significant ethical and data privacy issues.

Each of the three types of BCI targets different application scenarios, implementations, and technical challenges (McFarland and Krusienski, 2012; He et al., 2020). Therefore, the barriers faced by different types of BCI technologies exhibit significant differences. Unique barriers to invasive BCI: (1) Surgical risk. Invasive BCI requires surgical implantation of electrodes into the brain, which carries risks such as infection and bleeding; (2) Long-term stability. Tissue reactions to implanted electrodes may affect signal stability over time; (3) Biocompatibility. The biocompatibility of long-term implants presents a significant challenge, necessitating the use of materials that are stable and safe within living organisms. Unique barriers to semi-invasive BCI: (1) Signal quality and safety balance. Although semi-invasive BCI reduces the risk of invasive procedures, the signal quality is generally inferior to that of fully invasive BCI. Additionally, the long-term safety of the implantation site remains a concern; (2) Technology integration. Semi-invasive BCI must balance signal acquisition and user comfort. Unique barriers to non-invasive BCI: (1) Signal acquisition limitations. Non-invasive BCI signals weakened and more susceptible to external interference, must pass through the scalp and skull; (2) Device portability and comfort. Although surgery is not required, user acceptance of non-invasive BCI devices for extended use hinges on their comfort and portability; (3) Real-time and accuracy. Achieving high real-time and accurate decoding presents greater challenges for non-invasive BCI.

These barriers are also person-specific, but in a nutshell, they all boil down to a risk–benefit tradeoff. The aim of translating BCI into practical applications is to ensure that the benefits outweigh the risks, or that the advantages outweigh the disadvantages. It is important to acknowledge that all technologies carry risks and disadvantages. However, if the benefits outweigh the drawbacks, BCI technology can be considered without the need to excessively pursue perfection and overemphasize its potential. It is crucial to maintain objectivity and avoid biased language. For example, a terminally ill person in a locked-in state may be willing to undergo brain surgery even if BCI offers little or temporary benefit. On the contrary, somebody who is paralyzed from the neck down may not be willing to have brain surgery unless BCIs can restore function that is on par with what they can accomplish with residual motor control (sip-and-puff, eye tracker, speech recognition, and so on). By and large, the current BCIs fall short of the reliability and precision of residual control, but advances in machine learning or deep learning and large-scale recording may soon close this gap. For non-invasive BCIs, the risk is minimal but unwieldy equipment and relatively low performance may be a deal breaker for many applications.

Secondly, various paradigms such as MI, SSVEP, and P300 encounter common obstacles when translating BCI technology into practical applications, as well as unique challenges of their own (McFarland and Krusienski, 2012; Nicolas-Alonso and Gomez-Gil, 2012). Common obstacles: (1) User training requirements. BCI systems typically require users to undergo training before they can be used effectively, which restricts their immediate availability and widespread adoption. (2) System accuracy and stability. Improving the accuracy and stability of BCI systems remains a common challenge, particularly in dynamic and changing real-world environments. (3) Device portability and comfort. For extended wear and daily use, BCI devices often are bulky and uncomfortable. (4) Signal processing and decoding. Real-time and efficient signal processing and decoding algorithms are crucial for enhancing the performance of BCI systems. However, challenges still exist. (5) Individual differences. BCI systems require a high degree of personalization due to significant differences in brain signals among users.

Different paradigms of BCI face their unique barriers. For example, unique barriers to MI-BCI: (1) User training difficulty. MI-BCI requires significant training and concentration, posing challenges for some users to generate distinct MI signals; (2) Brain signal detectability. MI generates weak brain signals and is susceptible to interference from non-task-relevant brain activities. Unique barriers to SSVEP-BCI: (1) Visual fatigue. Prolonged viewing of flashing stimuli can lead to visual fatigue for the user, affecting the user experience and system performance, even with imperceptible flicker SSVEP-BCI (Ming et al., 2023); (2) External device dependency. SSVEP-BCIs rely on visual stimuli of specific frequencies and require external devices such as LEDs or displays to generate these stimuli, even when an augmented reality headset is used to provide the stimuli (Chen et al., 2020). Unique barriers to P300-BCI: (1) Variability of event-related potentials. The system’s accuracy may be affected by the variability of the P300 wave and the low signal-to-noise ratio; (2) Size limitation of the selection set. To ensure a high accuracy rate, the selection set (e.g., the alphabet of the speller) of the P300 BCI is often restricted. However, it limits the ITR.

To overcome these obstacles, it is necessary to conduct collaborative interdisciplinary research, involving joint efforts across various fields, including neuroscience, materials science, electrical engineering, computer science, and ethics.

## 5 Conclusion

To promote the translation of BCI into practical applications, the existing researches are reviewed, and the evaluation methods of the usability and user satisfaction of the entire online BCI system are detailed in the article, including the leap from analyzing and modeling for offline BCI data to the construction of online BCI systems and optimizing its performance, and comprehensive evaluation methods for translating BCI into practical applications (including the evaluation for usability, user satisfaction and usage of online BCI systems.). Finally, it is emphasized that the efficacy of BCI needs to be evaluated for specific end-users (user-centred) using the specific BCI system in specific application scenarios to achieve specific goals and that combining BCI with AI, VR, and other advanced technologies is essential to enhance the intelligence and user experience of the system. It is expected that this article will be useful for the development of BCI.

## Author contributions

HP: Writing – original draft, Investigation. PD: Writing – review & editing, Investigation, Conceptualization, Methodology, Supervision. FW: Investigation, Writing – review & editing, Methodology. TL: Investigation, Writing – review & editing. LZ: Investigation, Writing – review & editing, Funding acquisition. AG: Writing – review & editing. WN: Writing – review & editing. YF: Writing – review & editing, Supervision, Conceptualization, Funding acquisition.
